# Purinergic Receptors P2X7 and P2X4 as Markers of Disease Progression in the rd10 Mouse Model of Inherited Retinal Dystrophy

**DOI:** 10.3390/ijms232314758

**Published:** 2022-11-25

**Authors:** Natalia Martínez-Gil, Oksana Kutsyr, Agustina Noailles, Laura Fernández-Sánchez, Lorena Vidal, Xavier Sánchez-Sáez, Carla Sánchez-Castillo, Pedro Lax, Nicolás Cuenca, Antonio G. García, Victoria Maneu

**Affiliations:** 1 Departamento de Fisiología, Genética y Microbiología, Universidad de Alicante, 03690 Alicante, Spain; 2 Departamento de Óptica, Farmacología y Anatomía, Universidad de Alicante, 03690 Alicante, Spain; 3 Departamento de Farmacología y Terapéutica, Instituto-Fundación Teófilo Hernando, Facultad de Medicina, Universidad Autónoma de Madrid, 28029 Madrid, Spain; 4 Instituto de Investigación Sanitaria, Hospital Universitario de la Princesa, 28006 Madrid, Spain

**Keywords:** retinitis pigmentosa, purinergic receptors, retina, degeneration, inflammation, P2X7 receptor, P2X4 receptor

## Abstract

The purinergic receptor P2X7 (P2X7R) is implicated in all neurodegenerative diseases of the central nervous system. It is also involved in the retinal degeneration associated with glaucoma, age-related macular degeneration, and diabetic retinopathy, and its overexpression in the retina is evident in these disorders. Retinitis pigmentosa is a progressive degenerative disease that ultimately leads to blindness. Here, we investigated the expression of P2X7R during disease progression in the rd10 mouse model of RP. As the purinergic receptor P2X4 is widely co-expressed with P2X7R, we also studied its expression in the retina of rd10 mice. The expression of P2X7R and P2X4R was examined by immunohistochemistry, flow cytometry, and western blotting. In addition, we analyzed retinal functionality by electroretinographic recordings of visual responses and optomotor tests and retinal morphology. We found that the expression of P2X7R and P2X4R increased in rd10 mice concomitant with disease progression, but with different cellular localization. Our findings suggest that P2X7R and P2X4R might play an important role in RP progression, which should be further analyzed for the pharmacological treatment of inherited retinal dystrophies.

## 1. Introduction

Purinergic signaling is important in the development of inflammation associated with neurodegenerative diseases, and several purinergic receptors have emerged as potential therapeutic targets for degenerative diseases of the central nervous system (CNS). A major contributor to the inflammatory processes is the purinergic receptor P2X7 (P2X7R), which is involved in septic and sterile inflammatory diseases including viral and bacterial infections, but is also implicated in cancer and CNS diseases such as multiple sclerosis, Alzheimer’s (AD), and Parkinson’s (PD) disease [1,2,3,4,5,6,7,8,9].

P2X7R is broadly co-expressed with the P2X4 purinergic receptor; however, it remains unclear whether these subunits assemble to form functional heterotrimers, or whether they interact as homotrimeric receptors to form cooperative receptor complexes. Nonetheless, it has been suggested that a combination of both receptors participates in the processes of inflammation and nociception [10,11,12,13]. It has also been shown that these receptors can influence each other’s expression (reviewed in [11]). The role of P2X4R in neurodegeneration is less clear, although it is considered a central player in the pathogenesis of chronic and neuropathic pain and allodynia [14,15]. It is also involved in multiple pathologies including asthma, rheumatoid arthritis, Duchenne muscular dystrophy, brain tumors, and neurodegenerative diseases, including PD and amyotrophic lateral sclerosis [14,16,17,18,19,20,21,22].

Purines play a crucial role in the modulation of neural processing within the retina. The mammalian retina expresses most of the purinergic receptor subtypes, as well as the vesicular transporter of ATP (vesicular nucleotide transporter or VNUT), which packages purines into synaptic vesicles, and the molecular machinery required for ATP degradation [23,24,25]. Previous studies showed that P2X7R is expressed in astrocytes, microglia and Müller cells (the latter only in humans), in retinal pigment epithelium (RPE), and pericyte-containing retinal microvessels [25,26,27,28,29,30]. There is also a large body of evidence supporting the expression of P2X7R in neurons. Indeed, immunohistochemical studies have established the expression of P2X7R in the outer (OPL) and inner (INL) plexiform layers, in the inner nuclear layer (INL), and retinal ganglion cells (RGCs) [25,29,31,32,33]. Yet, the expression of P2X7R in neuronal cells has been questioned in recent years [34,35,36]. With respect to P2X4R, its expression overlaps with that of P2X7R, and both have been found in glial cells and also in horizontal cells, rod and cone bipolar cell terminals, and calretinin-positive amacrine cell bodies and processes [37].

Functionally, P2X7R and P2X4R are nonselective ligand-gated ion channels, activated by ATP and permeable to Na^+^, Ca^2+^, and K^+^. Under pathological conditions, stressed cells release large quantities of ATP that overstimulate P2X7R, which mediates the formation of plasma membrane pores, thereby inducing intracellular cytotoxic Ca^2+^ overload and activation of the inflammasome-dependent cell death pathway [38,39,40,41]. The ATP released by neurons or glial cells can induce, through P2X7R, microglial activation and proliferation, and the release of proinflammatory cytokines such as tumor necrosis factor-α and interleukin-1 beta. This, in turn, can promote further microglial activation in the retina and the propagation of retinal gliosis from a focal injury into the surrounding non-injured tissue, ultimately inducing secondary cell death [42,43] and worsening the neurodegenerative process [41,44]. While the role of P2X4R in pathological conditions is less understood, a relevant role for P2X4R in inflammatory diseases is becoming increasingly clear. P2X4R is known to trigger inflammation in response to high concentrations of extracellular ATP [21]. In this sense, it has recently been demonstrated that while microglial proliferation is mediated through P2X7R, ATP induces microglial migration through P2X4R [45].

With respect to the role of these receptors in retinal pathologies, previous studies have linked P2X7R to glaucoma [39,45,46], age-related macular degeneration (AMD) [2,16,47], and diabetic retinopathy [48,49,50]. In the case of retinal P2X4R, Ho et al. have suggested a role in modulating the neuronal function of photoreceptors and bipolar cells in the lateral inhibitory pathways, and also in glial signaling, tissue homeostasis, and immunosurveillance [37].

The main objective of the present study was to examine the expression of P2X7R and P2X4R during the progression of retinitis pigmentosa (RP), an inherited retinal dystrophy, using the rd10 mouse model. Our results indicate that the expression of both receptors increases with disease progression.

## 2. Results

### 2.1. Retinal Function in rd10 Mice

**Figure 1:**
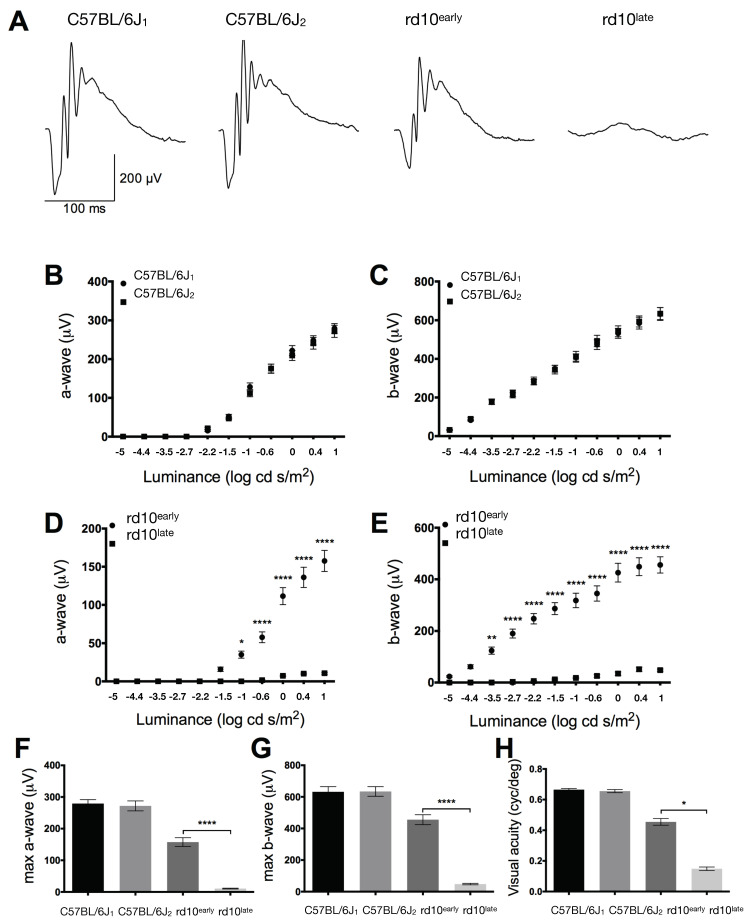
Effect of retinal degeneration on retinal function. (**A**) Representative dark-adapted ERG intensity responses to 1 log cd·s/m^2^ flashes in C57BL/6J, rd10early, and rd10late mice. (**B**–**E**) Luminance–response curves of C57BL/6J (**B**,**C**) and rd10 (**D**,**E**) mice (*n* = 3), showing measurements for a-wave (**B**,**D**) and b-wave (**C**,**E**) responses. (**F**,**G**): Mean values of a-wave (**F**) and b-wave (**G**) in the four groups of mice tested. (**H**): Visual acuity measured as the spatial frequency threshold in C57BL/6J, rd10early, and rd10late mice. Two-tail (ERG) and one-way (optomotor test) ANOVA and Tukey’s post hoc test. * *p <* 0.05, ** *p <* 0.01, **** *p <* 0.0001. C57BL/6J1: postnatal day (P)20; C57BL/6J2: P40; rd10early: P18 and rd10late: P40.

We first assessed the progressive loss of retinal functionality in rd10 mice through scotopic electroretinogram (ERG) responses (Figure 1A). ERG responses were lower in rd10 mice than in age-matched C57BL/6J control mice, and ERG recordings from rd10^late^ mice (late disease stage) showed very poor responsiveness (Figure 1A). The mean values of a- and b-wave amplitudes were similar between C57BL/6J_1_ (younger) and C57BL/6J_2_ (older) mice (Figure 1B,C, respectively), and were higher than those from rd10 mice (Figure 1D,E), with greater differences at higher values (44% decrease in rd10^early^ mice and 96% in rd10^late^ mice for the maximum a-wave, and 28% decrease in rd10^early^ and 92% in rd10^late^ for the maximum b-wave; Figure 1F,G) (*p <* 0.001). Also, the a- and b-wave amplitudes were smaller in rd10^late^ mice than in rd10^early^ mice (Figure 1D,E) (93% reduction in the maximum a-wave and 89% reduction in the maximum b-wave; Figure 1F,G) (*p <* 0.001).

We next evaluated visual acuity by optomotor testing. Visual acuity values were significantly lower in rd10^early^ and rd10^late^ mice than in age-matched C57BL/6J controls, confirming the degeneration of the retina of rd10 mice (32% and 77% lower, respectively), with no differences between the two C57BL/6J age groups (Figure 1H) (*p <* 0.05). As expected, visual acuity was significantly lower in rd10^late^ mice than in rd10^early^ mice (67% reduction).

### 2.2. P2X7R Expression in rd10 Mice

**Figure 2:**
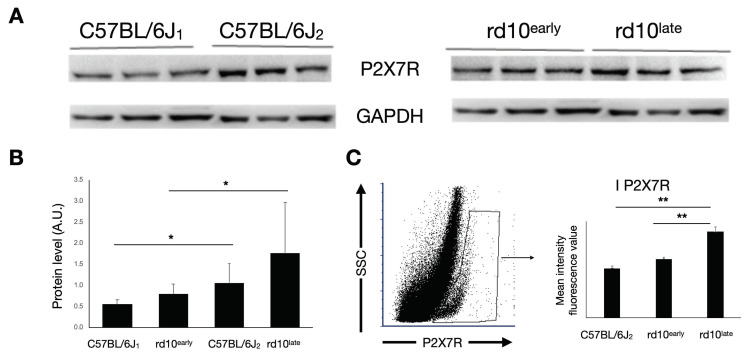
P2X7R expression in mouse retina. (**A**) Representative western blot and (**B**) protein quantification of P2X7R expression in C57BL/6J, rd10early, and rd10late mice. (**C**) Mean fluorescence values of P2X7R immunostaining in P2X7R-positive cells analyzed by flow cytometry in rd10early and rd10late mice compared with C57BL/6J mice. Student’s *t*-test * *p <* 0.05, ** *p <* 0.01. C57BL/6J1: postnatal day (P)20; C57BL/6J2: P40–P47; rd10early: P18 and rd10late: P44.

We next analyzed the expression of P2X7R in retinal cells of rd10 mice at early and later disease stages in comparison with its expression in control retinas of healthy age-matched C57BL/6J mice. Western blotting analysis revealed that P2X7R expression was significantly greater in rd10^late^ retinas than in both rd10^early^ and C57BL/6J_2_ retinas (Figure 2A,B) (*p <* 0.05). We confirmed this by flow cytometry, finding a significant increase in P2X7R immunofluorescence in rd10^late^ retinal cells (mean relative intensity value 4000 ± 300) relative to equivalent preparations from rd10^early^ (2700 ± 200) and C57BL/6J_2_ (2300 ± 300) mice (Figure 2C) (*p <* 0.01).

**Figure 3:**
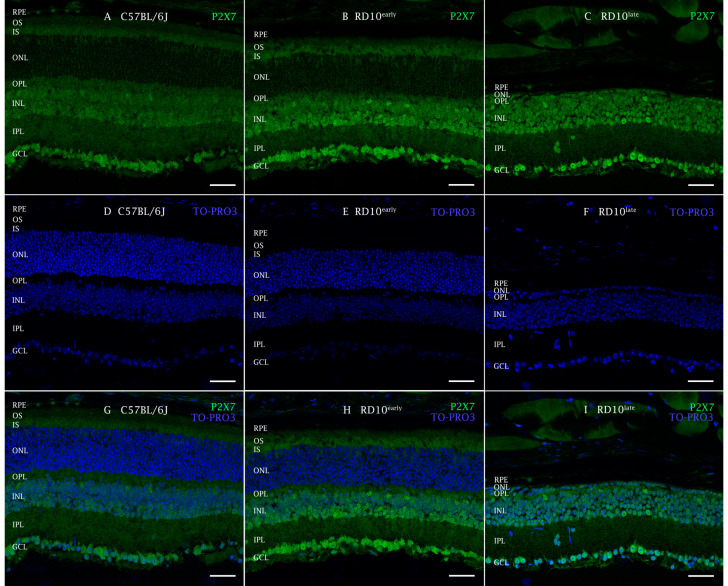
P2X7R expression in mouse retinas analyzed by immunohistochemistry. Cross-sectional cryosections of retinas from a representative C57BL/6J mouse (**A**,**D**,**G**) and rd10early (**B**,**E**,**H**) and rd10late (**C**,**F**,**I**) mice, stained with antibodies against P2X7R (**A**–**C**,**G**–**I**). Nuclei were stained with TO-PRO (**D**–**F**). In C57BL/6J mice, immunopositive fluorescence against P2X7R appears mainly located in the INL and GCL. RPE: retinal pigment epithelial cells; OS: outer segments; IS: inner segments; ONL: outer nuclear layer; OPL: outer plexiform layer; INL: inner nuclear layer; IPL: inner plexiform layer; GCL: ganglion cell layer. Scale bar: 50 µm.

Immunohistochemical analysis of retinal sections revealed a regular stippling pattern of P2X7R staining in all retinal layers, with greater immunoreactivity in ganglion cells and in the INL where horizontal amacrine and bipolar cells are located (Figure 3). Also, the inner segments of photoreceptors (cones and rods) presented robust immunoreactivity. The cell bodies of the cones also showed specific labeling.

### 2.3. P2X4R Expression in rd10 Mice

**Figure 4:**
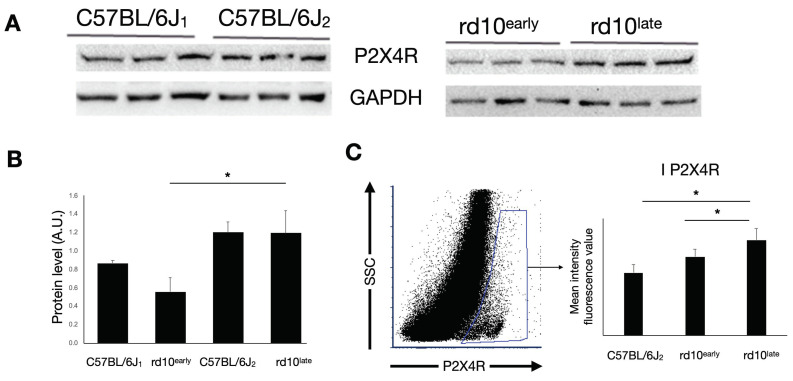
P2X4R expression in mouse retina. (**A**) Representative western blot and (**B**) protein quantification of P2X4R expression in C57BL/6J, rd10early, and rd10late mice. (**C**) Mean fluorescence values of P2X4R immunostaining in P2X7R-positive cells analyzed by flow cytometry in rd10early and rd10late mice compared with the control expression in C57BL/6J mice. Student’s *t*-test, * *p <* 0.05. C57BL/6J1: postnatal day (P)20; C57BL/6J2: P40-P47; rd10early: P18 and rd10late: P44.

Western blotting analysis showed that the expression of P2X4R was significantly higher in rd10^late^ retinas than in rd10^early^ and C57BL/6J_1_ retinas (Figure 4A,B) (*p* < 0.05). We also found a significant increase in P2X4R expression in rd10^late^ retinal cells by flow cytometry (mean intensity value 8000 ± 1000) as compared with equivalent cells from rd10^early^ (6700 ± 600) and C57BL/6J^late^ (5300 ± 700) retinas (Figure 4C) (*p* < 0.05).

**Figure 5:**
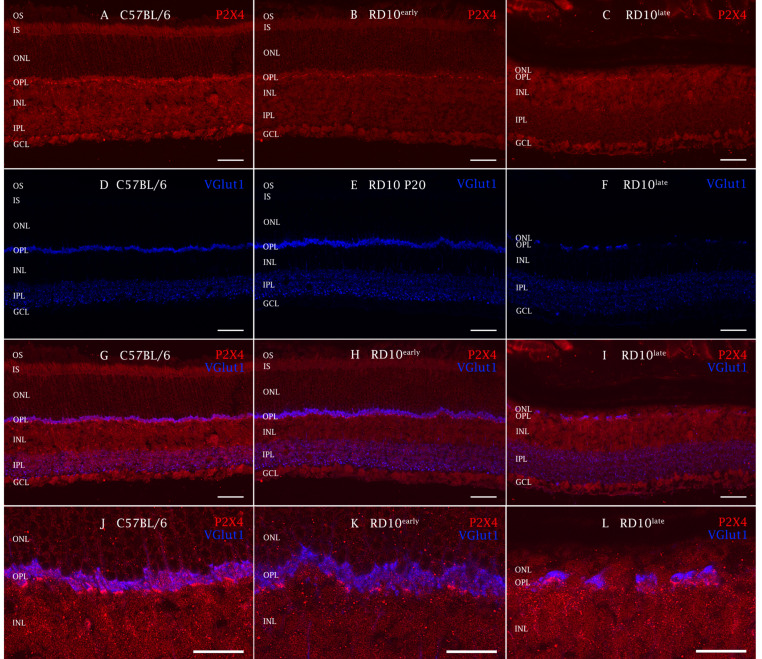
P2X4R expression in mouse retinas analyzed by immunohistochemistry. Cross-sectional cryosections of retinas from a representative C57BL/6J mouse (**A**,**D**,**G**,**J**) and rd10early (**B**,**E**,**H**,**K**) and rd10late (**C**,**F**,**I**,**L**) mice. Sections were stained with antibodies against P2X4R (**A**–**C**,**G**–**L**) and the vesicular glutamate transporter VGlut1 (**D**–**L**). Immunopositive areas of P2X4R appear in the terminal zone of the photoreceptors close to the VGlut1 immunoreactive zones (less noticeable in rd10late when most of the cones have disappeared) and GCL. RPE: retinal pigment epithelial cells; OS: outer segments; IS: inner segments; ONL: outer nuclear layer; OPL: outer plexiform layer; INL: inner nuclear layer; IPL: inner plexiform layer; GCL: ganglion cell layer. Scale bar: 50 µm. C57BL/6J: postnatal day (P)30; rd10early: P20 and rd10late: P40.

We observed that immunoreactivity to P2X4R in immunohistochemical preparations appeared in all groups tested as a regular stippling pattern throughout the retina (Figure 5) and was more evident in the photoreceptor terminals, close to the vesicular glutamate transporter 1 (VGlut1) immunoreactive zones, and in the INL, IPL, and the central ganglion layer.

### 2.4. CD11b-Positive Cells in Degenerating Retinas Overexpress P2X7R but Not P2X4R

To examine the implication of P2X7R and P2X4R in inflammatory response linked to degeneration, we used flow cytometry to assess their expression in the CD11b^+^ population, which includes microglia, monocytes, and other myeloid populations that actively participate in the inflammatory process.

**Figure 6:**
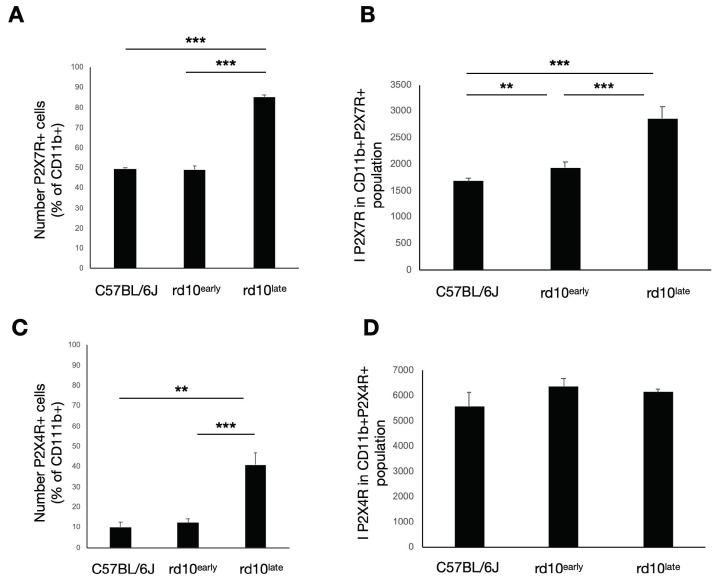
P2X7R and P2X4R expression in retinal myeloid cells (CD11b^+^). The CD11b immunoreactive cell population was analyzed by flow cytometry. Bar graphs show the number of CD11b^+^ cells expressing P2X7R (**A**) and the mean intensity of P2X7R fluorescence values (**B**). Also, the number of CD11b^+^ cells expressing P2X4R is shown (**C**), and the mean intensity of P2X4R fluorescence values (**D**). C57BL/6J: postnatal day (P)47; rd10early: P18 and rd10late: P44. ** *p <* 0.01, *** *p <* 0.001.

The subset of P2X7R-immunoreactive cells in the CD11b^+^ populations was similar in C57BL/6J_2_ (49.5 ± 0.6% of the CD11b population) and rd10^early^ (49 ± 2%) retinas, but was significantly higher in the retinas of rd10^late^ mice (85.2 ± 0.9%) (Figure 6A) (*p <* 0.001). Notably, the increase in the expression level of P2X7R in these cells was evident from the first stages of the degenerative process, as the mean fluorescence intensity value (I_P2X7R_) was significantly greater in rd10^early^ retinas (1940 ± 90) than in C57BL/6J_2_ retinas (1680 ± 50). As the degeneration evolved, the expression of P2X7R increased (2900 ± 200 for rd10^late^) (Figure 6B) (*p <* 0.01, *p <* 0.001).

Although the number of P2X4R-positive cells was significantly higher in the retinas of rd10^late^ mice (41 ± 6%), this population was not significantly different between rd10^early^ (12 ± 2%) and C57BL/6J_2_ (10 ± 3%) mice (Figure 6C) (*p <* 0.01, *p <* 0.001). Of note, in contrast to our observations for P2X7R, no significant differences were observed in the expression levels of P2X4R in any of the groups tested: I_P2X4R_ 5500 ± 600, 6300 ± 300, and 6100 ± 100 units for C57BL/6J_2_, rd10^early^ and rd10^late^, respectively (Figure 6D).

Overall, the results suggest that during the first stages of the degenerative process, when the myeloid-derived cell population remains stable, there is an early increase in the expression of P2X7R but not P2X4R in these cells. The expression of P2X7R increases as the degeneration evolves. In advanced stages, the subset of cells that express P2X7R and/or P2X4R increases, although the expression level of P2X4R in this subset of cells remains stable.

### 2.5. P2X7R and P2X4R Expression Levels Vary in Subsets of CD11b-Positive Cells

**Figure 7:**
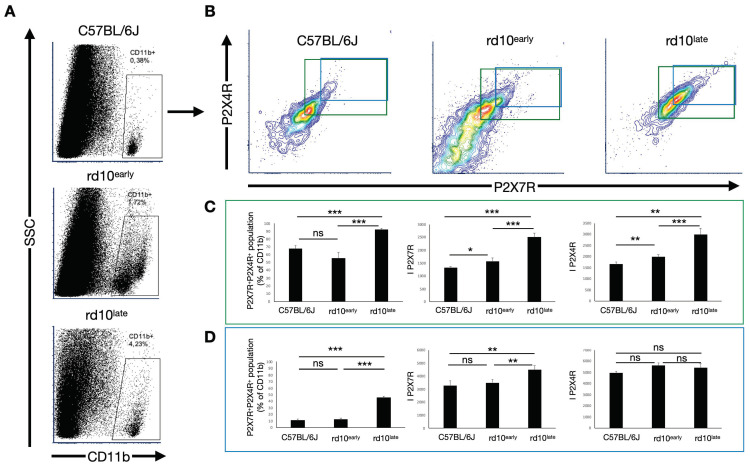
P2X7R and P2X4R expression in the CD11b^+^ population, analyzed by flow cytometry. Whole retinal cells from C57BL/6J, rd10early, and rd10late mice were triple-stained with antibodies against CD11b, P2X7R, and P2X4R. After discarding the cell debris and selecting singlets, CD11b^+^ cells were gated (**A**) and the expression of P2X7R and P2X4R was analyzed (**B**–**D**). (**B**) Double contour plots representing P2X7R and P2X4R expression, showing P2X7R- and P2X4R-positive populations (green line) and P2X7R- and P2X4R-highly immunoreactive populations (blue line). Each plot shows the sum of a minimum of 3 independent replicates. (**C**,**D**) Bar graphs showing the number of double-positive cells (P2X7R and P2X4R) and mean fluorescence values for P2X7R and P2X4R in the whole double-positive population (**C**) and the highly immunoreactive double-positive population (**D**). Student’s *t*-test, * *p <* 0.05, ** *p <* 0.01, *** p < 0.001, ns: non-statistically significant. C57BL/6J: postnatal day (P)47; rd10early: P18 and rd10late: P44.

As the expression levels of P2X7R and P2X4R appeared heterogeneous in the myeloid-derived cell population, we decided to analyze their expression levels in different cell subsets (Figure 7).

We first analyzed all CD11b^+^ cells with double immunoreactivity against P2X7R and P2X4R (Figure 7A–C). This population was more abundant in rd10^late^ mice (92 ± 2% of the CD11b^+^ population) than in rd10^early^ (56 ± 7%) and C57BL/6J (68 ± 4%) mice. In this cell subset, the expression levels of both P2X7R and P2X4R were increased from the first stages of the retinal degenerative process compared with healthy C57BL/6J retinas (I_P2X7R_: 1320 ± 30 in C57BL/6J mice, 1600 ± 100 in rd10^early^ and 2500 ± 100 in rd10^late^ mice; I_P2X4R_: 1650 ± 90 in C57BL/6J mice, 1980 ± 90 in rd10^early^ and 3000 ± 300 in rd10^late^ mice) (*p <* 0.05, *p <* 0.01, *p <* 0.001).

We could also observe a small CD11b^+^ population highly reactive for P2X4R and P2X7R (CD11b^+^P2X7R^hi^P2X4R^hi^) that was significantly greater in rd10^late^ mice (46 ± 5% of CD11b^+^ cells) than in rd10^early^ (13 ± 2%) or C57BL/6J (11 ± 2%) mice. Examination of this population revealed significant differences only in the expression levels of P2X7R in rd10^late^ mice, with no changes in the expression levels in rd10^early^, or in P2X4R expression at any age (I_P2X7R_: 3300 ± 400 in C57BL/6J, 3500 ± 200 in rd10^early^ and 4500 ± 300 in rd10^late^; I_P2X4R_: 4900 ± 200, 5600 ± 200 and 5400 ± 300 for retinas obtained from C57BL/6J, rd10^early^, and rd10^late^ mice, respectively) (Figure 7A,B,D) (*p <* 0.01, *p <* 0.001).

**Figure 8:**
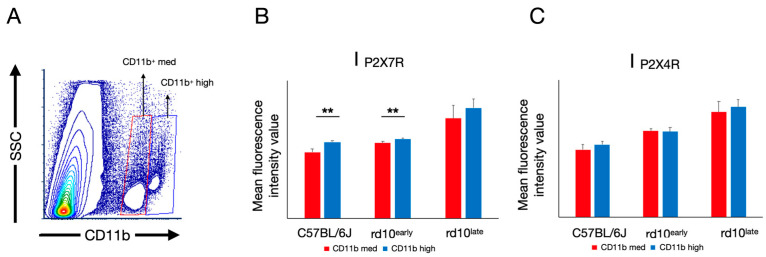
P2X7R and P2X4R expression analysis in CD11b+ cells, assessed by flow cytometry. Whole retinal cells from C57BL/6J, rd10early, and rd10late mice were triple-stained with antibodies against CD11b, P2X7R, and P2X4R. After discarding the cell debris and selecting singlets, CD11b-immunopositive cells were gated and two populations were selected according to medium or high immunoreactivity against the CD11b antibody, as shown in the contour plot (rd10 postnatal day [P]18) (**A**). The dot plot shows the sum of a minimum of 3 independent replicates. The immunoreactivity against P2X7R (**B**) and P2X4R (**C**) was compared in the two CD11b populations of each group of mice. Student’s *t*-test was performed between the two populations in each condition. Data are presented as mean values ± SD. Student’s *t*-test, ** *p <* 0.01. C57BL/6J: P47; rd10early: P18 and rd10late: P44.

In rd10^early^ mice, the CD11b^+^ population clearly showed two main phenotypes with regards to CD11b immunoreactivity (Figure 8): one showing medium reactivity against the CD11b antibody (CD11b^med^), and the other with high immunoreactivity (CD11b^hi^). The CD11b^hi^ population, which accounted for 0.04% of the total population in C57BL/6J mouse retinas, increased in number in both rd10^early^ (0.45%) and rd10^late^ (1.8%) mouse retinas. CD11b^hi^ and CD11b^med^ populations showed a similar mean fluorescence intensity value for P2X4R in each group of mice, with the mean I_P2X4R_ values for CD11b^hi^ vs. CD11b^med^: 3200 ± 200 vs. 3100 ± 300 (rd10^late^), 2500 ± 100 vs. 2500 ± 70 (rd10^early^), and 2100 ± 100 vs. 2000 ± 100 (C57BL/6J). The mean P2X7R fluorescence values in CD11b^hi^ vs. CD11b^med^ populations were significantly different in C57BL/6J^late^ (1940 ± 40 vs. 1690 ± 80) and rd10^early^ (2030 ± 30 vs. 1920 ± 40) mice (*p <* 0.01), but no significant differences were found in rd10^late^ mice (2800 ± 200 vs. 2500 ± 300). Overall, these results establish a population of CD11b^med^ cells that present with increased expression of P2X7R as degeneration evolves.

## 3. Discussion

In the present study in the rd10 mouse model of RP, we found that the loss of retinal function with age (i.e., from postnatal day 20 to 40) is paralleled by augmented expression of P2X7R. These findings suggest that P2X7R-mediated neuroinflammation is involved in disease progression, leading to loss of vision in RP.

A previous study showed that P2X7R is expressed in glial and neuronal cells as well as in the RPE [23]. We found that in rd10^late^ mice P2X7R immunoreactivity was more evident in RGC and INL. Curiously, flow cytometry analysis demonstrated that P2X7R expression was already enhanced in myeloid cells in rd10^early^ mice with respect to controls, including microglia and monocyte-derived macrophages. This suggests that P2X7R is involved in the early stages of inflammation; as retinal degeneration progresses, P2X7R expression further increases to strengthen inflammation. Our previous observations are consistent with this view, showing an increase in microglial cell numbers as retinal damage [51,52] and retinal neurodegeneration [53,54] develop. Furthermore, P2X7R seems to be central in microgliosis [7,43]. The finding that P2X7R is highly expressed in the small highly reactive population of CD11b cells (even in rd10^early^ mice) is in line with microgliosis and inflammation at early disease stages. Of note, a previous study found increased P2X7R expression in the degenerating retina of BALBCrds mice, another model of RP [55]; however, P2XR7 was not expressed in glial cells but the authors suggested that it could contribute to retinal damage.

Increased expression or overactivation of P2X7R has been described in different retinal degenerative diseases. In several models of glaucoma, a multifactorial retinal neurodegenerative disease, it has been shown that ATP released from activated Müller cells can trigger RGC apoptosis through P2X7R [39]. Also, increased extracellular levels of ATP and VNUT correlated with retinal dysfunction in glaucomatous retinas [56]. P2X7R has also been related to disease progression in diabetic retinopathy, as its stimulation or overexpression positively regulates the secretion of vascular endothelial growth factor and promotes angiogenesis [48]. Moreover, the susceptibility of retinal microvessels to P2X7R activation is enhanced in diabetes [49,50]. P2X7R also mediates AMD-like defects in vitro and in animal models of both dry and wet AMD [57].

We show that the expression of P2X4R in rd10 mice is mainly located in the INL, IPL, and RGC of retinas, and is more evident in the photoreceptor terminals in all groups tested. Increased P2X4R expression levels in rd10 mice were demonstrated at an advanced stage of the degenerative process. In myeloid cells, P2X4R expression did not change in the groups studied, although the subset of cells expressing this receptor increased in abundance. To date, the precise role of P2X4R in inflammatory processes is not understood. Our results point to a role for P2X4R in retinal degeneration, as its expression increases with the evolution of the disease and it is expressed in most of the cells expressing P2X7R, although its precise role remains to be elucidated.

Purinergic receptors have a complex role both in promoting and preventing inflammation. For instance, the potentiation of P2X4R signaling favors a switch to an anti-inflammatory phenotype in microglia [58], but P2X4R overexpression is also related to an upregulation of cytokines such as IL-6 and inflammation [14,59].

The presence of a subset of CD11b^+^ cells with a higher expression of P2X7R might contribute to the inflammatory state that quickly evolves in this mouse model. In agreement with our data, the presence of a highly reactive CD11b^+^ population has been observed in other disease models, for example, monocytes detected in the lung cells of an animal model of chronic obstructive lung disease correlate with inflammation [60]. We found a more heterogeneous population in an advanced stage of the degenerative process, as cells with different phenotypes develop. At this stage, at least some microglial cells increase their expression of CD11b along with other markers of cell activation. The different phenotypes appearing as degeneration evolves agree with our previous results [51,54,61,62].

The differences in the expression levels of P2X7R and P2X4R found in different subsets of cells and the differential changes in their expression as degeneration evolves indicate that both homomeric and/or heteromeric receptor combinations are possible. The possibility that the imbalance between P2X7R and P2X4R is a factor tilting the balance toward an inflammatory scenario remains to be determined.

The small CD11b^+^ population highly reactive against both P2X4R and P2X7R that significantly increases in rd10^late^ retinas could correspond to macrophages, as the expression of P2X7R in these cells can increase up to 10-fold [63,64]. Yu et al. proposed that monocyte-derived cells can accelerate degeneration [65], and a recent report from Funatsu et al. showed that the circulating inflammatory monocytes that reach the retina during degenerative processes and mediate cone cell death in rd10 mice [66]. Also, the co-expression of P2X4R and P2X7R has been demonstrated in macrophage invaders in other diseases such as Duchenne muscular dystrophy [17].

The available literature indicates that the modulation of P2X7R and P2X4R is important in pathologies involving inflammation. Indeed, purinergic antagonists have been found to be protective against cell death and degeneration [38,40,67,68,69,70], and the potentiation of P2X4R signaling by the allosteric modulator ivermectin favors a switch of microglia to an anti-inflammatory phenotype in a model of autoimmune encephalitis [58]. We acknowledge that a major limitation in the present study is the lack of functional analysis of the receptors in our model of RP; however, our results support the notion that pharmacological modulation of purinergic receptors could be of clinical therapeutic utility also in inherited retinal neurodegenerative diseases, as has been proposed in several other pathologies, and we consider the role of these receptors worthy of further study [2,41,44].

It must be remembered, however, that multiple cell death pathways are involved in retinal degeneration; thus, focusing on a single target may not be sufficient to delay the degenerative process. In this line, multitarget approaches have been successfully applied to the treatment of other chronic diseases such as cancer, acquired immunodeficiency syndrome, asthma, or cardiac failure [71,72,73,74]. Our results point to P2X7R and P2X4R as potentially suitable targets for the pharmacological treatment of inherited retinal dystrophies and the development of new therapies.

In conclusion, we show that the loss of retinal function with age in the rd10 mouse model of RP is paralleled by augmented expression of P2X7R, pointing to a role for this receptor in neuroinflammation and vision loss. Indeed, P2X7R and P2X4R could be important in pathologies involving inflammation as purinergic antagonists have shown protective effects against cell death and degeneration. The role of purinergic receptors should be further studied as potential targets in the pharmacological treatment of inherited retinal dystrophies.

## 4. Materials and Methods

### 4.1. Animals

The rd10 mouse model of RP was used at two stages of disease progression: an initial stage, postnatal day 18–20 (rd10^early^), and an advanced stage, postnatal day 40–44 (rd10^late^). Control C57BL/6J mice were matched for ages, at postnatal days 18–20 (C57BL/6J_1_) and 30–47 (C57BL/6J_2_). The study was conducted according to the guidelines of the Declaration of Helsinki and was approved by the Ethics Committee of the University of Alicante 2022/VSC/PEA/0022.

### 4.2. Electroretinography

We recorded scotopic ERG responses at postnatal days 18 and 40 in rd10 and control mice, as described [75]. After a period of adaptation to overnight darkness, procedures were carried out to prepare the animals for bilateral ERG recording under dim red light. Animals were anesthetized with ketamine (100 mg/kg, i.p.) and xylazine (4 mg/kg), and they were kept on a heating pad at 38 °C. Pupils were dilated with a topical application of 1% tropicamide (Alcon Cusí, Barcelona, Spain). One drop of Viscotears 0.2% polyacrylic acid carbomer (Novartis, Barcelona, Spain) was placed on the cornea to facilitate electrical contact with the recording electrodes and to prevent dehydration. Dawson Trick Litzkow (DTL) fiber electrodes were used, consisting of an X-Static silver-coated nylon conductive strand (supplied by Sauquoit Industries, Scranton, PA, USA). The reference electrode was a 25-gauge platinum needle placed between the eyes under the scalp. Anesthetized animals were placed in a Faraday cage and all experiments were conducted in total darkness. Scotopic flash-induced ERG responses to light stimuli produced by a Ganzfeld stimulator were recorded in both eyes. Light stimuli were administered at increasing luminance (ranging from −5.2 to 0 log cd·s/m^2^) for 10 ms each. The mean of 3–10 consecutive recordings was calculated for each light exposure. An interval of 10 s was provided between flashes for dim flashes (−5.2 to −1.4 log cd·s/m^2^) and as much as 20 s for higher luminance (−0.8 to 0 log cd·s/m^2^). ERG signals were first amplified and then band-pass filtered (1–1000 Hz, without notch filtering) by means of a DAM50 data acquisition board (World Precision Instruments, Aston, UK). A PowerLab system (AD Instruments, Oxfordshire, UK) was used for the administration of stimuli and data acquisition (4 kHz).

### 4.3. Optomotor Test

The spatial frequency threshold was assessed for awake, freely moving C57BL/6J and rd10 mice at postnatal days 18 and 45. The Argos system (Instead, Elche, Spain) was used to observe and score optomotor responses to horizontally drifting, vertically oriented gratings. The spatial frequency threshold for the behavior was considered to be the maximum spatial frequency at maximum contrast that was still capable of inducing smooth head-tracking movements. For this test, the mouse was positioned on a platform in the center of a chamber whose sides were four computer monitors. Sinusoidal gratings were projected on all monitors as a virtual cylinder centered on the head and rotating in both horizontal directions. An overhead infrared video camera was used by a trained observer to record the mouse and score smooth head turns in response to the rotating gratings. These tracking responses were observed to be robust at middle spatial frequencies and diminished until they disappeared at the threshold.

### 4.4. Immunohistochemistry

Immunohistological studies of the retinas were performed with samples obtained from C57BL/6J mice at postnatal day 30 and rd10 mice at postnatal days 20 and 40, following well-established procedures [76]. Briefly, the animals were euthanized in the morning, the eyes were enucleated and fixed in 4% (*w/v*) paraformaldehyde for 1 h at room temperature (RT). After washing in 0.1 M phosphate buffer pH 7.4 (PB), the eyes were sequentially cryoprotected in 15, 20, and 30% (*w/v*) sucrose. The cornea, lens, and vitreous body were removed, and the eyecups were embedded in Tissue-Tek OCT (Sakura Finetek, Zoeterwouden, The Netherlands). Sixteen-micrometer-thick sections were obtained using a cryostat, mounted on slides (Superfrost Plus; Menzel GmbH and Co. KG, Braunschweig, Germany) and stored at −20 °C. Before further use, slides were thawed, washed 3 times with PB, and incubated with blocking solution (10% (*v/v*) donkey serum and 0.5% (*v/v*) triton X-100 in PB) for 1 h. Sections were subjected to single or double immunostaining with combinations of antibodies at different dilutions in PB with 0.5% Triton X-100 overnight at RT.

Primary antibodies used in this work have been extensively used in previous studies and have been well characterized regarding cell type specificity. Sections were first incubated with one or more of the following primary antibodies: rabbit anti-P2X7 receptor (extracellular) (1:200; Alomone Labs, Jerusalem, Israel), rabbit anti-P2X4 receptor (extracellular) (1:200; Alomone Labs), and guinea pig anti-vesicular glutamate transporter 1 (VGluT1) (1:1000; Chemicon-Millipore, Billerica, MA, USA). On the next day, retinal sections were subsequently washed in PB and incubated with the corresponding mixture of the following secondary antibodies at a 1:100 dilution for 1 h at RT: AlexaFluor 488–anti-rabbit IgG donkey and AlexaFluor 633–anti-guinea pig IgG donkey (Invitrogen, Carlsbad, CA, USA). TO-PRO 3-iodide (Invitrogen) was added at 1 μM with the secondary antibodies to visualize nuclei. Images were taken with a Leica TCS SP8 confocal laser-scanning microscope (Leica Microsystems, Wetzlar, Germany). Images were processed in parallel with Adobe Photoshop 10 software (Adobe Systems Inc., San Jose, CA, USA).

### 4.5. Flow Cytometry

Expression of P2X7R and P2X4R in retinal cells was analyzed using flow cytometry in C57BL/6J mice at postnatal day 47 and in rd10 mice at postnatal days 18 and 44. Eyes were enucleated, and the retinas were dissected, placed in 1 mL of PB, and disaggregated by gently pipetting up and down through a wide bore pipette tip. The resulting cell suspension was filtered through a 30-µm strainer (BD Biosciences, San Diego, CA, USA) to avoid cell clumps. The retinal cell suspension was then triple-stained with a cocktail of the following antibodies: rabbit anti-P2X7 receptor-ATTO633 (extracellular) (1:200; Alomone Labs), Rabbit anti-P2X4 receptor-FITC (extracellular) (1:200; Alomone Labs), and anti-CD11b-PE (Clone M/170; e-Bioscience, San Diego, CA, USA). Each mouse retina was individually analyzed. Data were acquired on an LSR Fortessa cytometer (BD Biosciences) and analyzed using FCS 6 Flow Cytometry Software (De Novo, Los Angeles, CA, USA).

### 4.6. Western Blotting

Protein extracts from C57BL/6J mice at postnatal days 20 and 40 and from rd10 mice at postnatal days 18 and 44 were obtained and resolved by denaturing sodium dodecyl sulfate-polyacrylamide gel electrophoresis (SDS-PAGE). Retinas were isolated, and proteins were extracted using RIPA buffer (Sigma-Aldrich, Madrid, Spain) containing protease (complete EDTA-free; Roche, Mannheim, Germany) and phosphatase (PhoStop; Roche) inhibitor cocktails. After incubation on ice for 30 min, debris was pelleted by a 10-min centrifugation step (10,000× *g*) at 4 °C and protein concentration in the supernatants was quantified with the Bio-Rad Protein Assay (Bio-Rad, Philadelphia, PA, USA) using bovine serum albumin as standard. Then, 20 µg of proteins was diluted in Laemmli sample buffer (4% SDS, 100 mM dithiothreitol, 20% glycerol, 0.004% bromophenol blue, 125 mM Tris-HCl, pH 6.8) and resolved on 5–12% SDS-PAGE gels. Proteins were transferred onto PVDF membranes (Roche), and the blots were blocked with 2.5% (*w/v*) non-fat dry milk in Tris-buffered saline/0.1% Tween-20 (TBS/T, pH 7.6), for 1 h at RT. Membranes were incubated at 4 °C overnight with the appropriate dilution of the following primary antibodies: rabbit anti-P2X7 receptor (extracellular) (1:200; Alomone Labs), rabbit anti-P2X4 receptor (extracellular) (1:200; Alomone Labs), and mouse anti-GAPDH (Sigma-Aldrich). After three 5-min washes with TBS/T, blots were incubated with the corresponding peroxidase-conjugated secondary antibody (ThermoFisher Scientific, Waltham, MA, USA) for 1 h at RT. Blots were then washed again three times with TBS/T, briefly rinsed with PB, and developed with the Lumi-light western blotting substrate (Roche).

### 4.7. Statistical Analysis

Data for C57BL/6J and rd10 mice were statistically analyzed using Prism software (GraphPad Software, Inc., San Diego, CA, USA). The D’Agostino and Person normality test was applied to determine whether the variables followed a normal distribution. A multivariate analysis of variance (MANOVA) with Bonferroni’s post hoc test was applied to assess significant differences in ERG values. Student’s *t*-tests were conducted to evaluate visual acuity and variables from western blotting and flow cytometry. Data are reported as the mean ± SD. Values of *p* < 0.05 were considered to be statistically significant.

## Data Availability

Not applicable.
